# Activating transcription factor 6 is involved in the beneficial effects of growth hormone-releasing hormone antagonists in the corneal endothelium

**DOI:** 10.3389/fopht.2026.1946815

**Published:** 2026-09-30

**Authors:** Md Matiur Rahman Sarker, Saikat Fakir, Madan Sigdel, Nektarios Barabutis

**Affiliations:** School of Basic Pharmaceutical and Toxicological Sciences, College of Pharmacy, University of Louisiana Monroe, Monroe, LA, United States

**Keywords:** endothelium, growth hormone, inflammation, keratitis, unfolded protein response

## Abstract

The corneal endothelium maintains eye transparency through fluid regulation, and its dysfunction has been associated with impaired vision. This study is focused on the possible involvement of activating transcription factor 6 (ATF6) in the beneficial effects of Growth Hormone - Releasing Hormone antagonists (GHRHAnt) in corneal cells. ATF6 is a UPR sensor involved in barrier function, previously shown to exert anti-inflammatory activities. The aim of this study is to evaluate involvement of ATF6 in the effects of GHRHAnt in the corneal endothelium. MTT assay was used to measure the cell viability of ATF6 modulator. We demonstrated the efficient ATF6 manipulation by AA147 and Ceapin-A7, which are specific ATF6 modulators. Western blot analysis was used to detect the protein expression levels. FITC-dextran assay and DCFDA were utilized to measure paracellular permeability and reactive oxygen species generation, respectively. AA147 enhanced Grp94 and BiP expression, both being downstream ATF6 targets, whereas Ceapin-A7 exerted the opposite effects. Then, we investigated the possible involvement of ATF6 in the protective activities of GHRHAnt in the corneal endothelium. ATF6 activation enhanced the protective effects of GHRHAnt by reducing paracellular permeability and ROS generation, while ATF6 inhibition exerted the opposite effects. Our study suggests that ATF6 mediates - at least in part - the beneficial effects of GHRHAnt in the cornea.

## Introduction

Unfolded protein response (UPR) is a critical cellular element that regulates endoplasmic reticulum (ER) homeostasis during stress conditions (1). Activation of UPR sensors - namely protein kinase RNA-like ER kinase (PERK), inositol‐requiring transmembrane kinase 1α (IRE1α), and activating transcription factor 6 (ATF6) - triggers adaptive responses through augmentation of molecular chaperones, reduction of new protein synthesis, and initiation of protein degradation (2). UPR is crucial for preserving endothelial barrier integrity (3) and prevents endothelial impairment (4). However, prolonged ER stress can interrupt endothelial homeostasis, contributing to intercellular junction disruption and cytoskeletal remodeling; leading to barrier dysregulation (4, 5).

ATF6 plays a pivotal role in regulating endothelial function (6). During ER stress, it migrates to the Golgi for cleavage, and regulates the expression of BiP and Grp94, which are molecular chaperones (7) that enhance protein-folding capacity and facilitate cell viability (8). Moreover, ATF6 regulates the stability and turnover of junctional proteins (6) and triggers protective adaptation and barrier integrity restoration. On the other hand, ATF6 depletion impairs junctional integrity and cellular stress responses (9, 10).

This molecular dysregulation has significant implications for ocular health, especially in keratitis (11). The corneal endothelium maintains corneal transparency through active fluid regulation and stromal dehydration (12). Endothelial impairment – particularly from ER stress-mediated inflammation - leads to stromal swelling and corneal clarity loss (13). In severe keratitis, endothelial defense impairment can lead to permanent corneal scarring and vision loss (13, 14). Therefore, understanding the crosstalk between endothelial permeability and UPR activation in the context of ATF6, could be considered critical for developing potential therapeutic strategies to mitigate corneal endothelial dysfunction.

Growth hormone–releasing hormone antagonists (GHRHAnt) exert anti-inflammatory responses in a diverse variety of tissues - and the endothelium - and suppress GH signaling. Several GHRHAnt, including JV-1-36, have been commonly utilized in research (15) and have been associated with anti-inflammatory and cytoprotective effects (16). Recent findings suggest that JV-1–36 alleviates LPS-induced inflammation in endothelial corneal cells by suppressing endothelial hyperpermeability, reactive oxygen species (ROS) generation, mitigating MAPK and STAT activation, and reducing pro-inflammatory cytokine production (17).

In this study, we examined the effects of GHRHAnt in cells exposed to ATF6 modulators, namely Ceapin-A7 or AA147, to investigate the possible role of ATF6 in mediating the effects of GHRHAnt in the corneal endothelium. It was previously shown that ATF6 is involved in endothelial barrier regulation and protects against inflammation (18–21). To the best of our knowledge, this is the first study to address the effects of ATF6 modulation in the effects of GHRHAnt in corneal endothelial cells.

## Materials and methods

### Reagents

VWR (Radnor, PA) provided DMSO (25-950-CQC), anti-rabbit IgG HRP secondary antibody (95017-556), RIPA buffer (AAJ63306‐AP), 2′,7′-dichlorofluorescein diacetate (10180-506), nitrocellulose membranes (10063-173), anti-mouse IgG HRP-linked whole antibody from sheep (95017-554) 3-(4,5-Dimethylthiazol-2-yl)-2,5-diphenyltetrazolium bromide (MTT) (BT142015–5G), and Corning trans-well cell culture inserts (29442-084). JV-1-36 (031-23) was purchased from Phoenix Pharmaceuticals Inc., located at Burlingame, CA. Grp94 (2104S) and BiP (3183S) antibodies were obtained from Cell Signaling Technology, which is in Danvers, MA. Tocris (Bristol, UK) and Abcam (Waltham, MA, USA) provided AA147 (6759) and protease inhibitor (AB287909), respectively. Sigma-Aldrich (St. Louis, MO) delivered FITC-Dextran (46945), β-actin antibody (A5441), and Ceapin-A7 (SML2330-5MG).

### Cell culture and treatment

ATCC (Manassas, VA, USA) provided bovine corneal endothelial cells (BCE C/D-1b) (CRL-2048), which were grown in DMEM (Dulbecco’s Modified Eagle Medium) enriched with 1% streptomycin/penicillin, 10% fetal bovine serum, and 1% amphotericin B per provider’s instructions (22). The dosage of ATF6 modulators were selected based on past observations (18–21).

### MTT assay and western blot analysis

Bovine corneal endothelial cells were seeded on a 96-well plate (10,000 cells in each well) and were treated with either AA147 or Ceapin-A7. After treatment, cell viability was measured using MTT solution following a previously established protocol (23). Western blot analysis was used for the quantification of protein expression utilizing a previously established protocol (24).

### Paracellular permeability and reactive oxygen species generation assay

Fluorescein isothiocyanate (FITC)–dextran permeability assay was utilized for the measurement of paracellular permeability. FITC–dextran (70 kDa, 1 mg/mL) solution was added to the trans-well inserts for 45 minutes. Then, 100 µL of culture medium was collected from the designated wells, and fluorescence intensity was measured using a Synergy H1 Hybrid Multi-Mode Reader (BioTek, Winooski, VT, USA). For ROS assessment, cells were seeded in 96-well plates at a density of 1 × 10^4^ cells per well. After treatment, the cells were incubated with 2′,7′-dichlorofluorescein diacetate (25 μM) for 45 minutes, and fluorescence intensity was measured utilizing the settings previously described.

### Densitometry and statistical analysis

Images obtained with the Bio-Rad ChemiDoc System (Hercules, CA) were analyzed using the National Institute of Health ImageJ software. The densitometric data were expressed as Mean ± SD (Standard Deviation) and the proteins of interest were normalized to β-actin. Statistical analysis was employed using GraphPad Prism (version 5.01). Statistical analysis was carried out by using one‐way analysis of variance (ANOVA), followed by Newman-keuls *post hoc* in multiple comparison test (**Figure 1**) and Student’s t-test (**Figures 2**–**4**). Differences were considered statistically significant at P-value <0.05. The number *n* in the figure legends represents the number of independent experiments.

**Figure 1:**
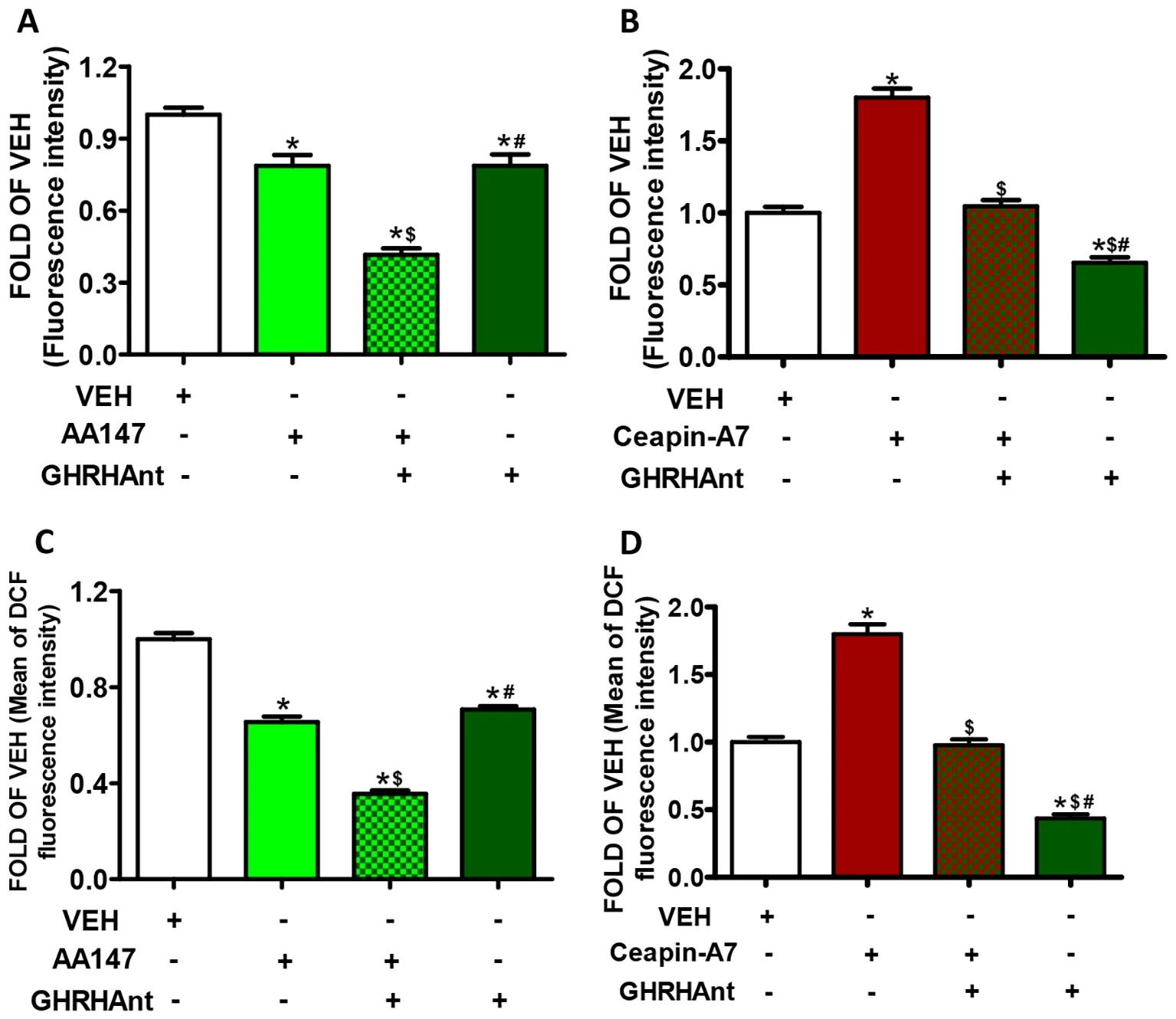
Assessment of paracellular permeability utilizing FITC-Dextran in BCE-C/D-1b treated with vehicle (0.1% DMSO) or **(A)** AA147 (10 μM) and **(B)** Ceapin-A7 (15 μM) for 24 hours and post-treated with VEH (0.1% DMSO) or JV-1-36 (1 μM) for 8 hours. Assessment of ROS generation in BCE-C/D-1b were treated with vehicle (0.1% DMSO) or **(C)** AA147 (10 μM) and **(D)** Ceapin-A7 (15 μM) for 24 hours and post-treated with VEH (0.1% DMSO) or JV-1-36 (1 μM) for 8 hours. ^*^P < 0.05 vs. VEH, ^$^P < 0.05 vs. AA147, and *^#^*P < 0.05 vs. AA147+GHRHAnt **(A, C)**; ^*^P < 0.05 vs. VEH, ^$^P < 0.05 vs. Ceapin-A7, and *^#^*P < 0.05 vs. Ceapin-A7+GHRHAnt **(B, D)**. The graphs represent **(A, B)** five independent experiments (n = 5), **(C)** twelve independent experiments (n = 12) and **(D)** eight independent experiments (n = 8). Data are expressed as Means ± SEM.

**Figure 2:**
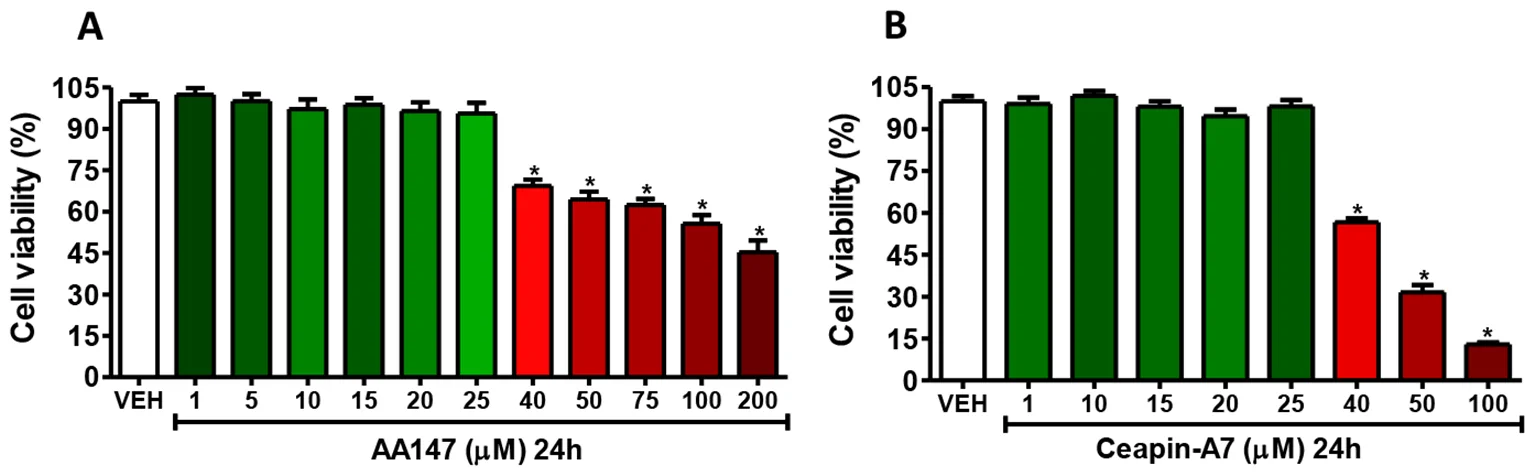
BCE-C/D-1b cells were treated with either vehicle (VEH) (0.1% DMSO) or **(A)** AA147 (1, 5, 10, 15, 20, 25, 40, 50, 75, 100, and 200 μM) for 24 hours; **(B)** Ceapin-A7 (1, 10, 15, 20, 25, 40, 50, and 100 μM) for 24 hours. Cell viability was evaluated by MTT assay. ^*^P < 0.05 vs VEH, **(A)** n = 6 and **(B)** n = 5. Means ± SEM.

**Figure 3:**
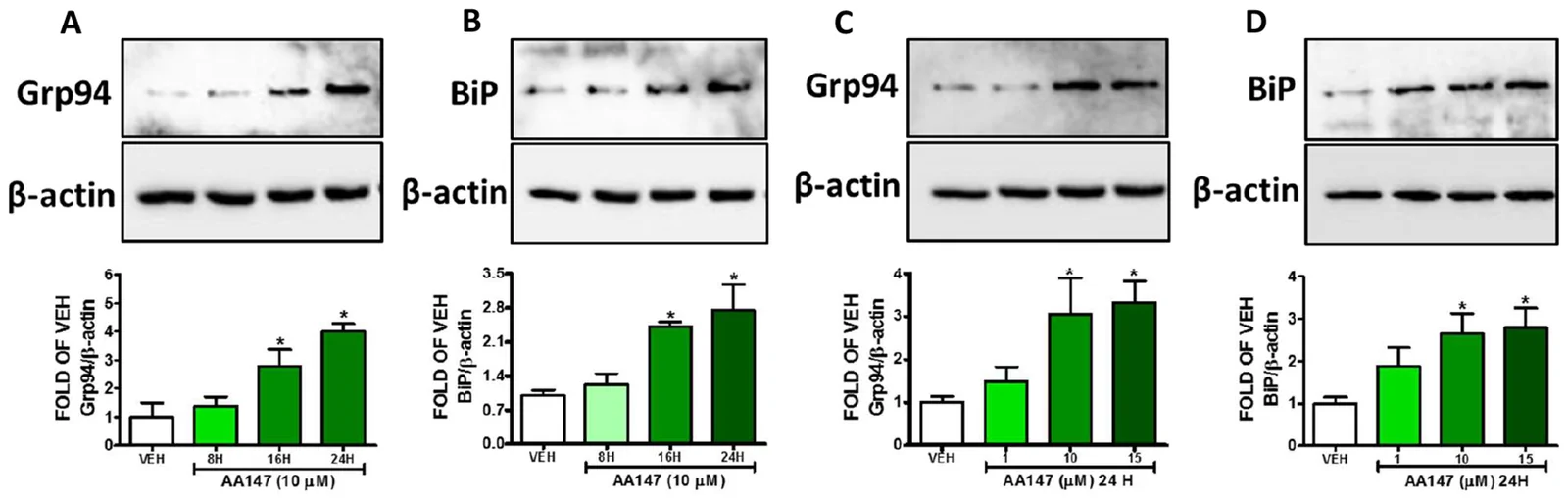
Western Blot analysis of **(A)** Grp94 and β-actin;**(B)** BiP and β-actin after exposure of BCE-C/D-1b to AA147 (10 μM) for 8, 16, and 24 hours. ^*^P < 0.05 vs VEH. Western Blot analysis of **(C)** Grp94 and β-actin; **(D)** BiP and β-actin after exposure of BCE-C/D-1b to AA147 (1, 10, and 15 μM) for 24 hours. ^*^P < 0.05 vs VEH. In all panels, β-actin was used as a loading control. Signal intensity was analyzed by densitometry. n = 3. Means ± SEM.

**Figure 4:**
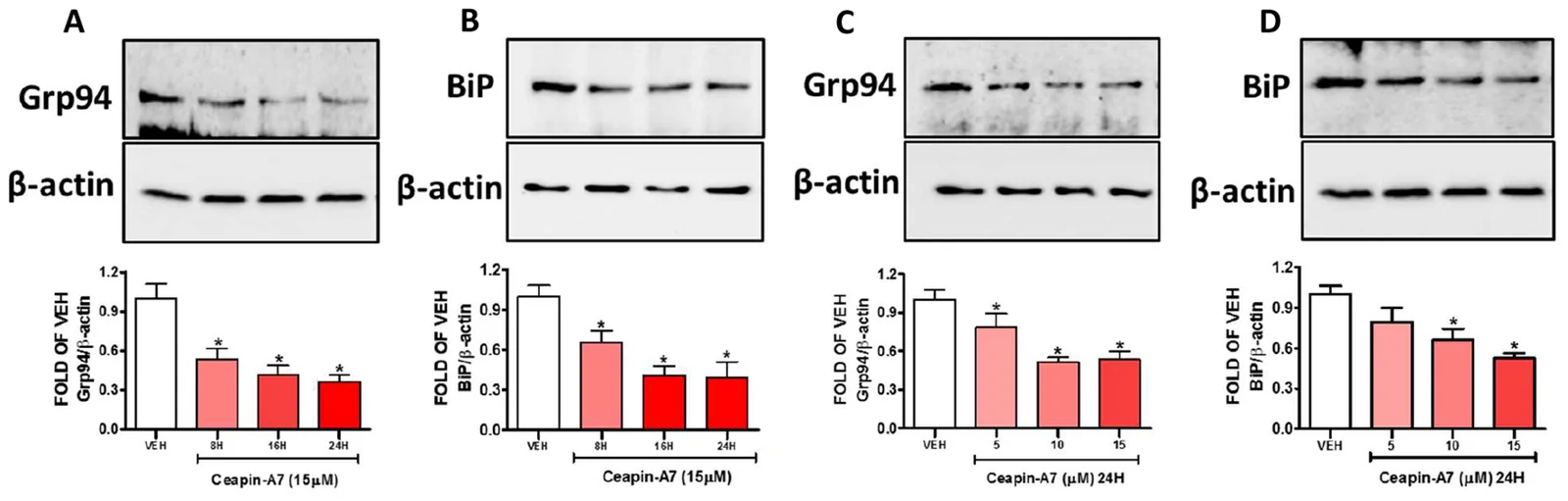
Western Blot analysis of **(A)** Grp94 and β-actin; **(B)** BiP and β-actin after exposure of BCE-C/D-1b to Ceapin-A7 (15 μM) for 8, 16, and 24 hours. ^*^P < 0.05 vs VEH. Western Blot analysis of **(C)** Grp94 and β-actin; **(D)** BiP and β-actin after exposure of BCE-C/D-1b to Ceapin-A7 (5, 10, and 15 μM) for 24 hours. ^*^P < 0.05 vs VEH. For all panels, signal intensity was analyzed by densitometry. β-actin was used as a loading control. n = 3. Means ± SEM.

## Results

### Effects of AA147 and Ceapin-A7 on cell viability

First, we assessed cell viability using MTT assay to observe the effects of (**Figure 2A**) AA147 (1-200 μM) and (**Figure 2B**) Ceapin-A7 (1–100 μM) on BCE-C/D-1b cells after being treated for 24 hours at different concentrations; per previous studies (18–21). Cell viability was not affected at the concentration range of 1–25 μM.

### AA147 induces ATF6 activation - related proteins in corneal endothelial cells

Next, we assessed the expression levels of Grp94 and BiP in BCE-C/D-1b cells due to AA147 exposure at (**Figures 3A, B**) different periods (8, 16, and 24h) and (**Figures 3C, D**) doses (1, 10, 15 μM). AA147 treatment induces ATF6 activation in BCE-C/D-1b cells by upregulating the expression of Grp94 and BiP in both cases. Grp94 is a downstream target gene activated by the transcription factor ATF6 (25). BiP is a molecular chaperone and its overexpression increases ATF6/BiP complex formation and delays the cleavage of ATF6 in response to ER stress (26).

### Ceapin-A7 inhibits ATF6 in corneal endothelial cells

We observed the effects of ATF6 inhibition in BCE-C/D-1b cells after being exposed to different doses of Ceapin-A7 (**Figure 4**). Our data suggest that Ceapin-A7 suppresses ATF6, since it downregulates Grp94 and BiP (**Figure 4**).

### ATF6 modulates the effects of GHRHAnt in corneal endothelial cells

In BCE-C/D-1b cells, GHRHAnt improved the integrity of endothelial barrier, and this effect was further potentiated by the ATF6 inducer AA147. Cells were pretreated with AA147 before GHRHAnt administration (**Figure 1A**). Both compounds produced a significant reduction in paracellular permeability, indicating improved barrier function. Notably, the combined treatment resulted in a greater decrease in paracellular permeability than either agent alone (18).

Pharmacological inhibition of ATF6 with Ceapin-A7 attenuated the barrier-protective effects of GHRHAnt. In BCE-C/D-1b cells, pretreatment with Ceapin-A7 before GHRHAnt exposure (**Figure 1B**) significantly increased paracellular permeability compared with cells treated with GHRHAnt alone, indicating impaired barrier integrity.

While GHRHAnt treatment alone reduced endothelial permeability and preserved barrier integrity, Ceapin-A7 promoted paracellular permeability and diminished the protective effects of GHRHAnt (20). FITC-dextran permeability assay demonstrates that ATF6 plays a key role in regulating endothelial barrier function and paracellular permeability. Collectively, these findings highlights ATF6 as a mediator in vascular protection.

### ATF6 modulates reactive oxygen species suppression due to GHRHAnt

In corneal endothelial cells, AA147 and GHRHAnt decrease the generation of ROS, indicating that both substances have anti-oxidative properties. In BCE-C/D-1b (**Figure 1C**), compared to AA147 or GHRHAnt therapy alone, ATF6 induction before GHRHAnt exposure led to a larger decrease in ROS production (18). In contrast, Ceapin-A7 works against ROS reduction. GHRHAnt had antioxidative effects in BCE-C/D-1b (**Figure 1D**), but ATF6 inhibition led to a rise in ROS production (20). Our observations implicate ATF6 as a regulator of the endothelial response to GHRHAnt, particularly in controlling oxidative stress. Because excessive ROS generation is a major determinant of endothelial dysfunction, inflammation, tissue injury, and impaired barrier function.

## Discussion

The cornea, a transparent and avascular connective tissue, consists of five layers - Bowman’s layer, epithelium, Descemet’s membrane, stroma, and endothelium - contributing to refractive function and visual transparency (27). The outermost epithelium protects from pathogens and environmental insults by serving as a shield. Bowman’s layer, beneath the epithelium, involves structural support but exhibits minimal regenerative potential (28). The stroma, composed of keratocytes and collagen fibers, contributes to the majority of corneal thickness (about 90%) and is responsible for ensuring transparency (29, 30).

Descemet’s membrane, the basement membrane for endothelium, engages in maintaining structural integrity, corneal homeostasis, and clarity. The innermost endothelium, a hexagonal cell monolayer lining the posterior surface, is crucial for upholding stromal deturgescence through a “pump–leak” mechanism to maintain corneal transparency. It regulates the transportation of ions and fluid between the anterior chamber and stroma (27, 31, 32).

Corneal endothelial cells (CEC) have very limited proliferative capacity due to the involvement of multiple factors, including negative cell cycle regulators, cell-cell contact inhibition, and growth inhibitors (33). The mean CEC density in neonatal is about 3000–4000 cells/mm^2^, which is reduced by 0.6% per year; and declined to 2500 cells/mm^2^ and 2000 cells/mm^2^ in adulthood and old age, respectively (33). Fuchs Endothelial Corneal Dystrophy (FECD), inflammation, and intraocular surgical trauma exacerbate CEC reduction (34). Thus, this decline in cell density and cumulative oxidative or inflammatory insults can result in a reduction of barrier integrity and pump function, leading to corneal edema and vision impairment (35, 36).

Corneal infections, including keratitis, represent a vital cause of corneal morbidity globally and result from viral, bacterial, fungal, or parasitic pathogens. Among other factors, wearing contact lenses remains a major risk factor, mostly due to microtrauma, hypoxia, and inadequate hygiene that trigger microbial adherence and growth (37, 38). Pathogen-associated molecular patterns stimulate immune responses in CEC, which trigger the inflammatory pathway, leading to endothelial barrier disruption, resulting in endothelium hyperpermeability, swelling of stroma, and reduced corneal transparency (39, 40).

Existing pharmacological strategies to treat corneal inflammation include antibiotics, antifungals, antivirals, and anti-inflammatory drugs such as corticosteroids and nonsteroidal anti-inflammatory drugs (NSAIDs). These treatment options have serious limitations, including secondary infections development, wound healing delay, and increased intraocular pressure, though they are effective in controlling inflammation (41, 42). So, there is a need for pharmacological approaches that will restore barrier stability with diminishing adverse effects.

Emerging evidence highlights the role of the UPR in regulating corneal endothelial and epithelial barrier function. In corneal endothelial cells, ER stress has been linked to impaired permeability (43, 44), and persistent stress in diabetic mice can cause corneal edema and endothelial cell loss (45). UPR activation is regulated by three transmembrane proteins, including protein kinase RNA-like ER kinase (PERK), inositol‐requiring transmembrane kinase 1α (IRE1α), and activating transcription factor 6 (ATF6) (46). PERK reduces new protein synthesis by phosphorylation of eukaryotic translation initiation factor two subunit-α (eIF2α), and IRE1α is involved in translocation and degradation of misfolded ER protein by activating X-box-binding protein 1 (XBP1) and regulated IRE1-dependent decay (RIDD) (47).

ATF6 plays a substantial role in restoring barrier stability among UPR branches. It is localized to the Golgi apparatus, and is cleaved by membrane transcription factors, site-1 protease (S1P) and site-2 protease (S2P) to produce an active transcription factor, ATF6p50. This activated transcription factor triggers the upregulation of chaperones such as BiP and Grp94, leading to the enhancement of protein folding capacity, and also facilitates the biogenesis of the Golgi apparatus and ER to augment secretory capacity of the cells, which contributes to restoring ER proteostasis. This adaptive response preserves barrier integrity and promotes cell viability (47, 48). ATF6 exhibits anti-inflammatory properties and also contributes to maintaining retinal homeostasis and photoreceptor development (49). However, loss of ATF6 leads to different diseases, including achromatopsia, cochlear damage (50), diabetes (51), heart disease, and obesity (52, 53).

ROS generation exacerbates corneal endothelial barrier disruption through intensification of ER stress and inflammation. Excessive ROS generation can lead to cytoskeletal contraction, junction disruption, and redox-sensitive signaling (e.g., NF-κB and MAPK) activation (54, 55). Notably, UPR pathways and ROS are firmly interconnected. While oxidative stress can aggravate ER stress, impaired UPR can contribute to ROS augmentation. This bidirectional crosstalk forms a pathological feedback loop that accelerates endothelial impairment and barrier disruption (56–58).

In this study, we reveal that modulation of ATF6 significantly influences corneal endothelial function in BCE-C/D-1b cells. At the concentration range of 1–25 μM (**Figure 2**), both AA147 and Ceapin-A7 did not exhibit toxic effects, validating their utilization in the study. According to our data, AA147 effectively triggered the ATF6 activation, as evidenced by the augmentation of Grp94 and BiP expression levels (**Figure 3**), whereas Ceapin-A7 downregulated the ATF6 signaling (**Figure 4**). Functionally, the activation of ATF6 enhanced the barrier-protective effects of GHRHAnt (**Figure 1A**), while ATF6 inhibition reversed these benefits and increased barrier permeability (**Figure 1B**). Our findings also indicate that AA147 potentiated GHRHAnt-mediated reduction of ROS generation (**Figure 1C**), whereas Ceapin-A7 treatment reversed that effect (**Figure 1D**). The aforementioned findings underscore the importance of ATF6-dependent signaling in regulating corneal endothelial integrity. Previous studies have already reported that GHRHAnt alleviate experimental ocular inflammation (59) induce apoptosis in retinoblastoma cells (60) and protect against LPS-induced corneal endothelial inflammation (17). Furtermore, it was previously shown that ATF6 is involved in barrier function and integrity (6, 61), and the endothelial effects of GHRHAnt are affected by UPR both *in vitro* (62), as well as *in vivo* (63). The efficacy of both Ceapin-A7 and AA147 towards ATF6 cleavage has been previously reported (6).

In conclusion, our study reports for the first time that that ATF6 is involved in the mediation of the protective effects of GHRHAnt in corneal endothelial paracellular permeability. Activating ATF6 may provide a promising therapeutic avenue for alleviating inflammatory corneal disorders such as keratitis, which can be due to bacteria or contact lenses use. Interestingly, somatostatin analogs, which have been FDA-approved, have been shown to activate ATF6, but they could not be considered targeted ATF6 activators.

Future studies will validate our findings in human corneal endothelial cells, will focus on an *in vivo* study on experimental models of rabbit corneal dysfunction to validate the *in vitro* findings, as well as to assess ATF6 cleavage to directly reinforce pathway specificity. Furthemore, off-target effects of ATF6 modulation will be investigated on stress pathways such as PERK-elF2alpha, since they can affect barrier function (64–66).

## Data Availability

The original contributions presented in the study are included in the article/**Supplementary Material**. Further inquiries can be directed to the corresponding author.
