# Supplementary material for: Activating transcription factor 6 is involved in the beneficial effects of growth hormone-releasing hormone antagonists in the corneal endothelium
Source: Front Ophthalmol (Lausanne). 2026 Sep 30;6:1946815. doi: 10.3389/fopht.2026.1946815 (PMC13661439; doi:10.3389/fopht.2026.1946815)
Supplement: Supplementary file 1 [file DataSheet1.pdf]

Supplementary Data

Figure 3A

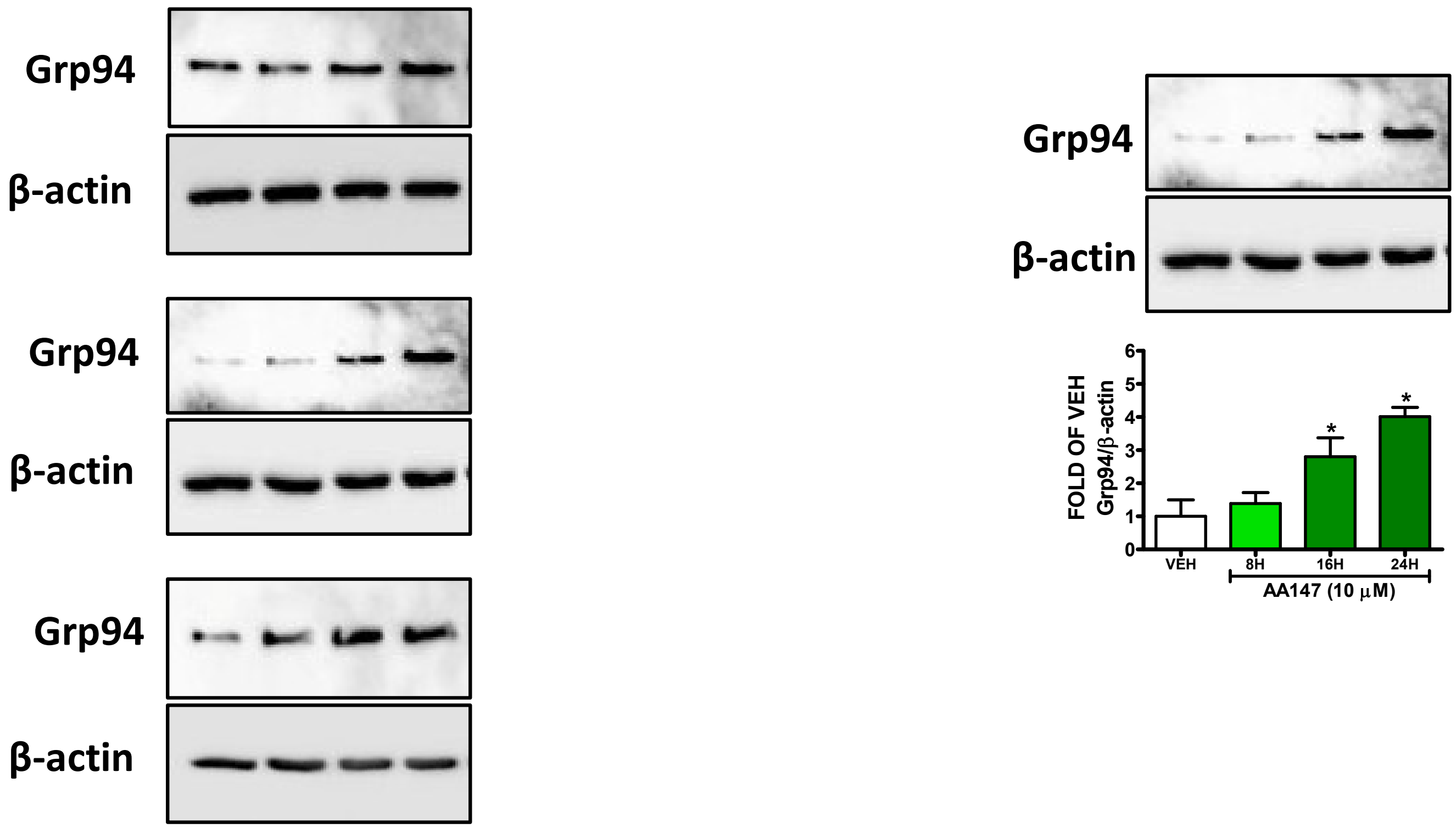
Grp94
β-actin
Grp94
β-actin
Grp94
β-actin
Grp94
β-actin
FOLD OF VEH
Grp94/β-actin
0
1
2
3
4
5
6
*
*
VEH
8H
16H
24H
AA147 (10 μM)

Figure 3A

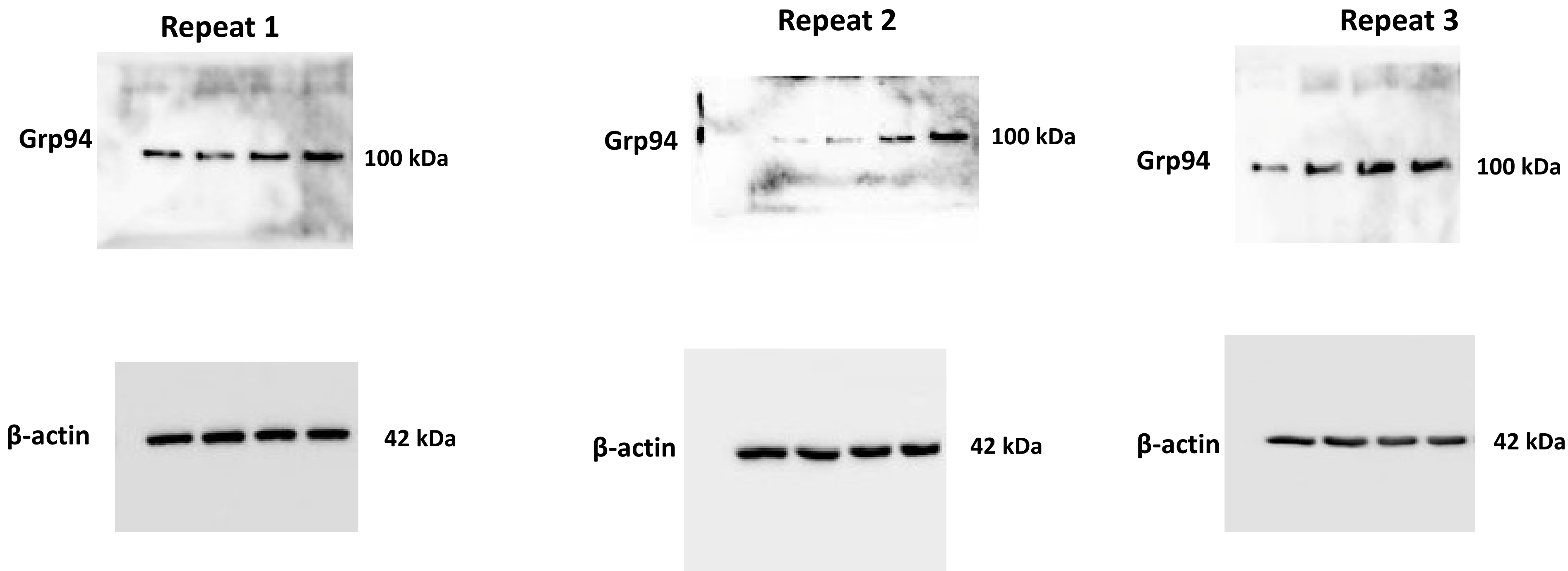

Repeat 1
Grp94
100 kDa
β-actin
42 kDa
Repeat 2
Grp94
100 kDa
β-actin
42 kDa
Repeat 3
Grp94
100 kDa
β-actin
42 kDa

Figure 3B

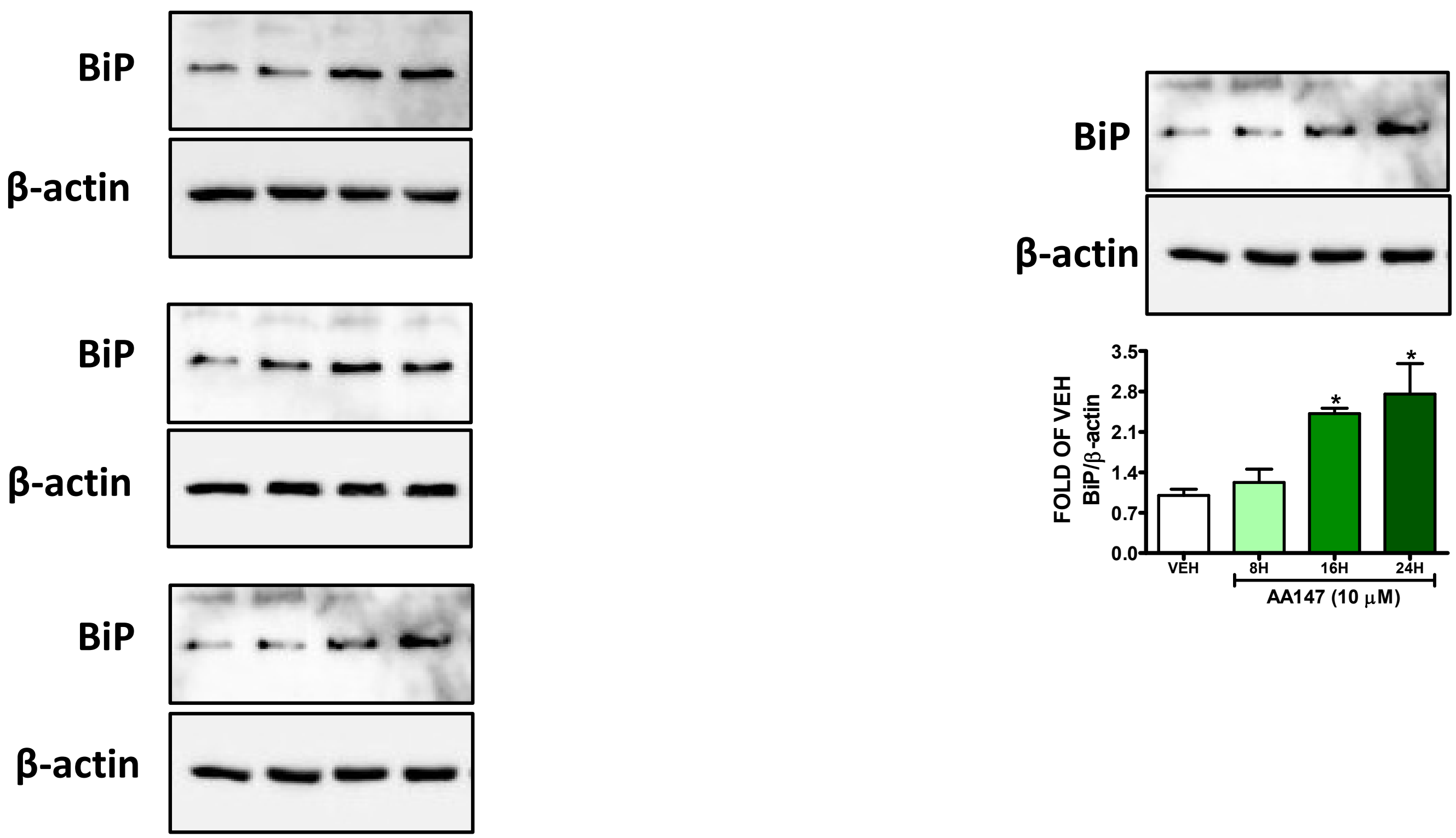
BiP
β-actin
BiP
β-actin
BiP
β-actin
BiP
β-actin
FOLD OF VEH
BiP/β-actin
3.5
2.8
2.1
1.4
0.7
0.0
*
*
VEH
8H
16H
24H
AA147 (10 μM)

Figure 3B

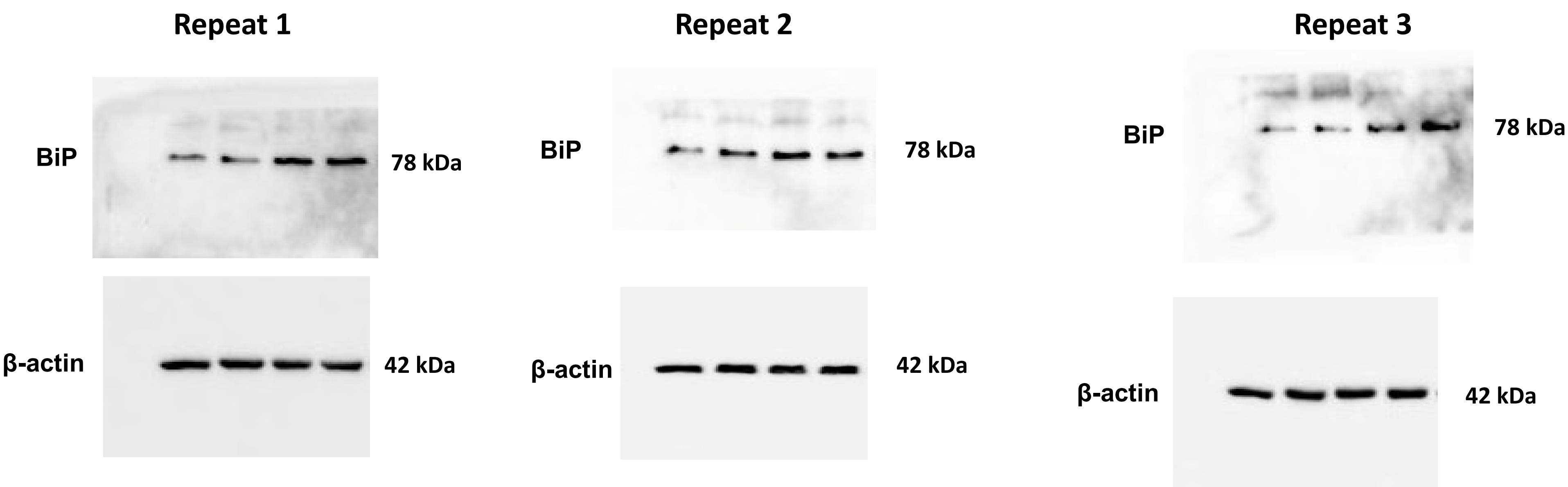
Repeat 1
BiP
78 kDa
β-actin
42 kDa
Repeat 2
BiP
78 kDa
β-actin
42 kDa
Repeat 3
BiP
78 kDa
β-actin
42 kDa

Figure 3C

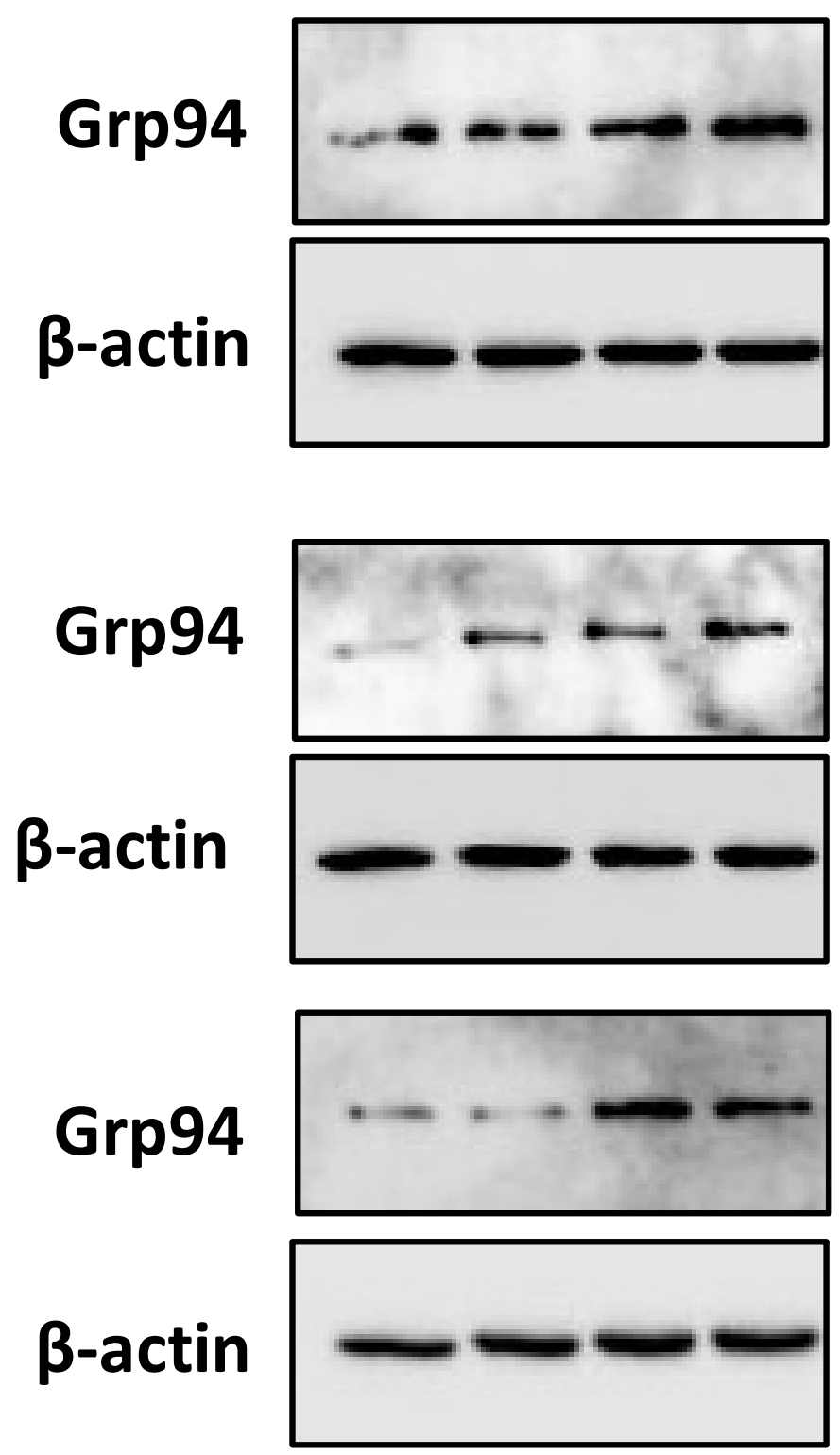
Grp94
β-actin
Grp94
β-actin
Grp94
β-actin

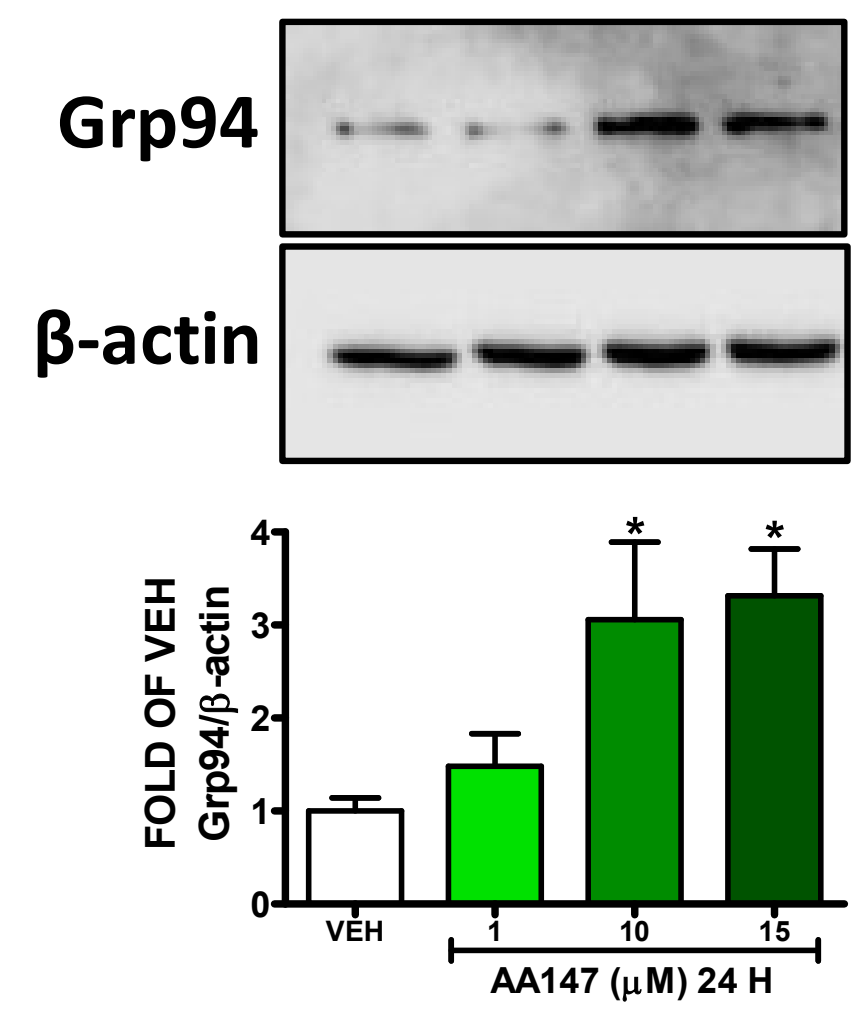
Grp94
β-actin
FOLD OF VEH
Grp94/β-actin
0
1
2
3
4
*
*
VEH
1
10
15
AA147 (μM) 24 H

Figure 3C

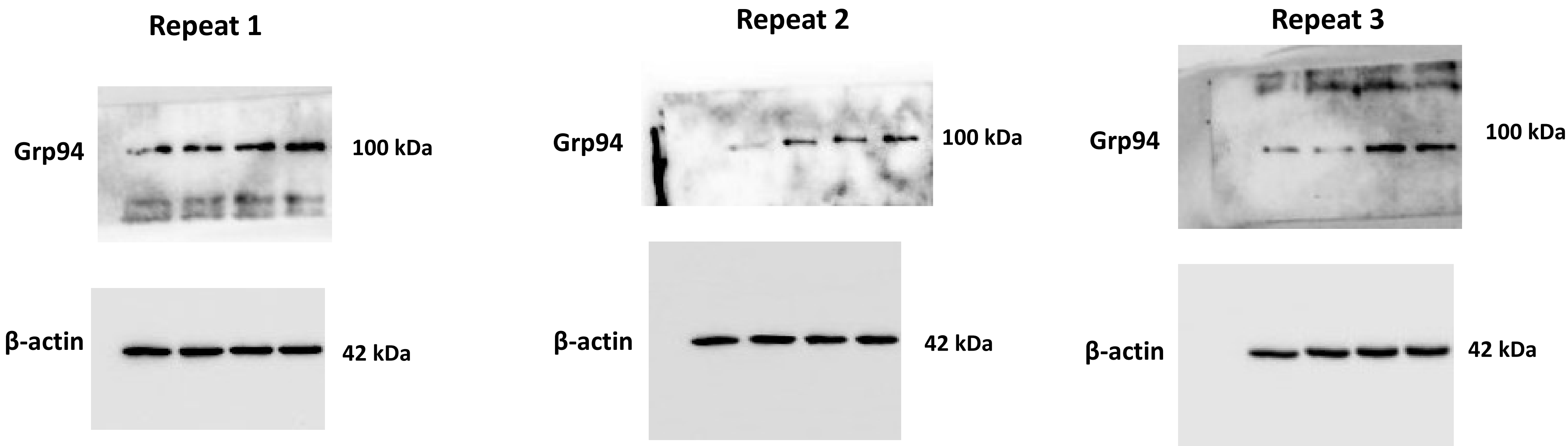
Repeat 1
Grp94
100 kDa
β-actin
42 kDa
Repeat 2
Grp94
100 kDa
β-actin
42 kDa
Repeat 3
Grp94
100 kDa
β-actin
42 kDa

Figure 3D

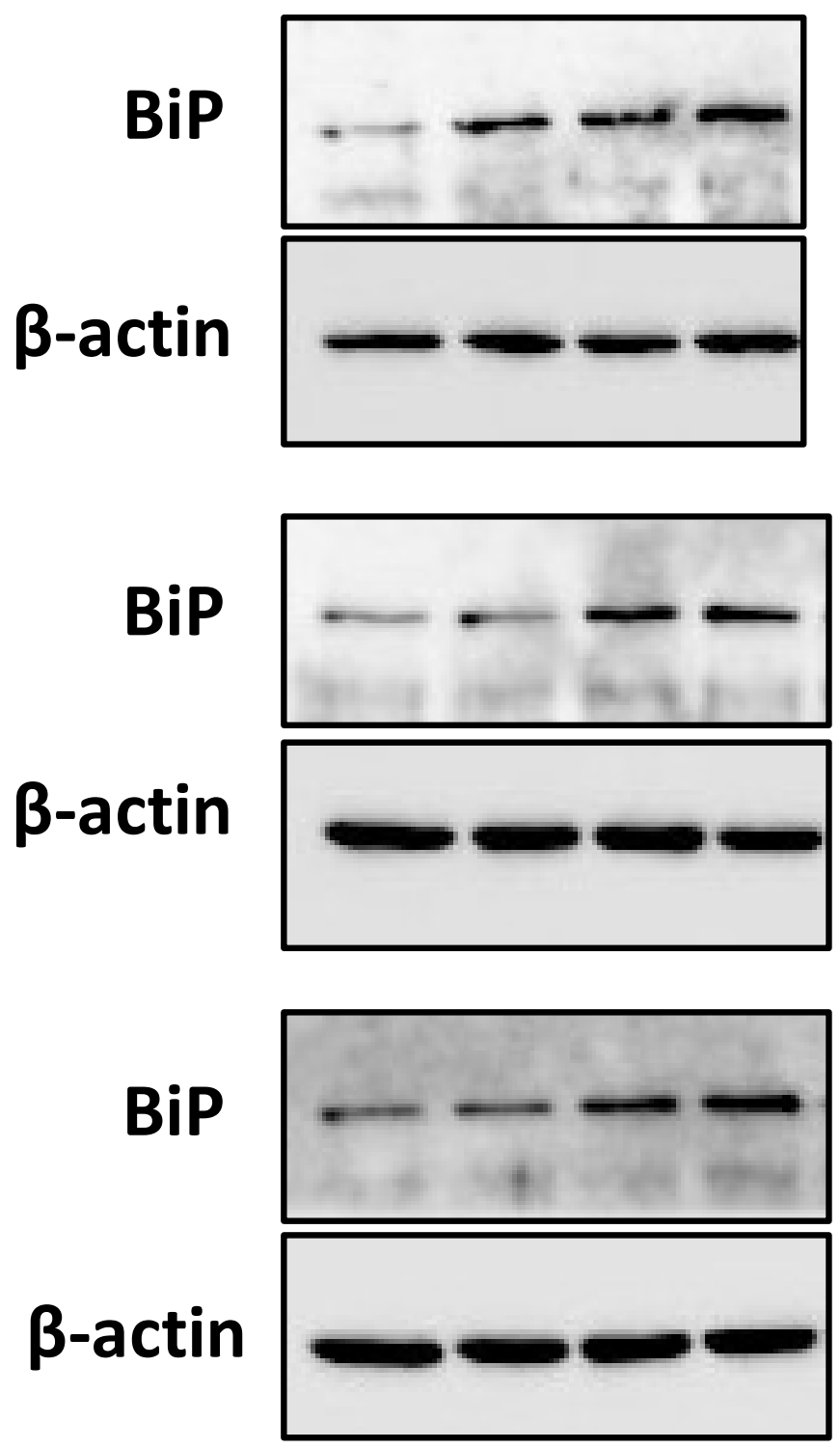

BiP
β-actin
BiP
β-actin
BiP
β-actin

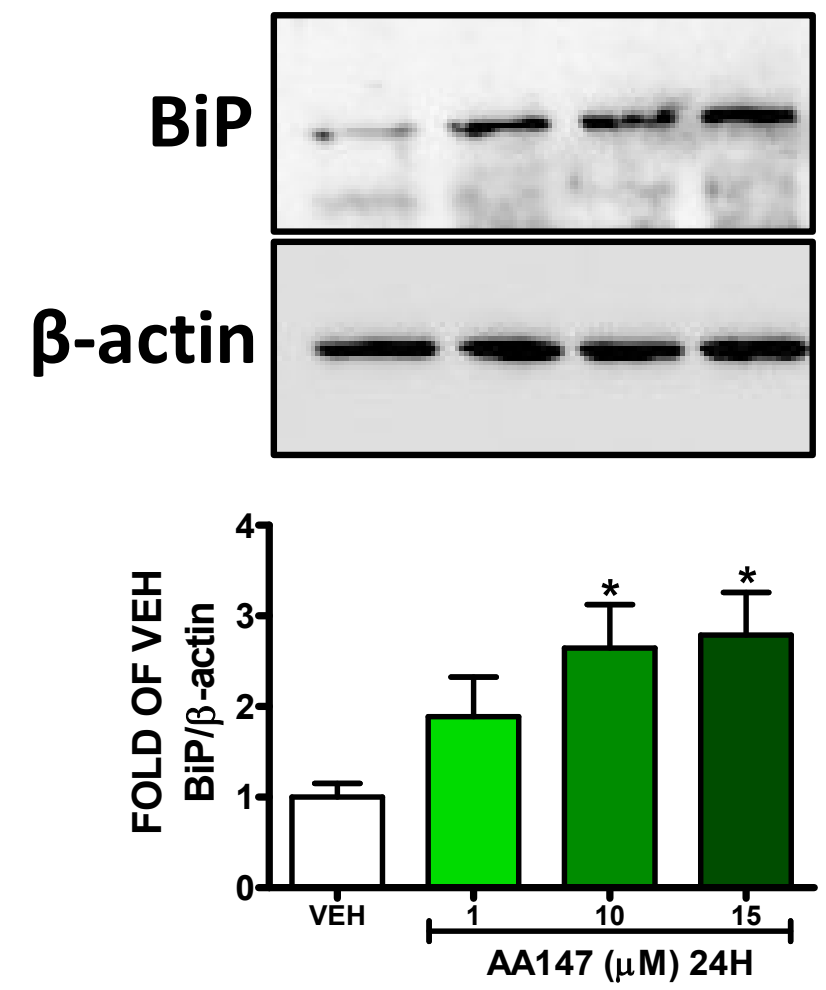

BiP
β-actin
FOLD OF VEH
BiP/β-actin
4
3
2
1
0
*
*
VEH
1
10
15
AA147 (μM) 24H

Figure 3D

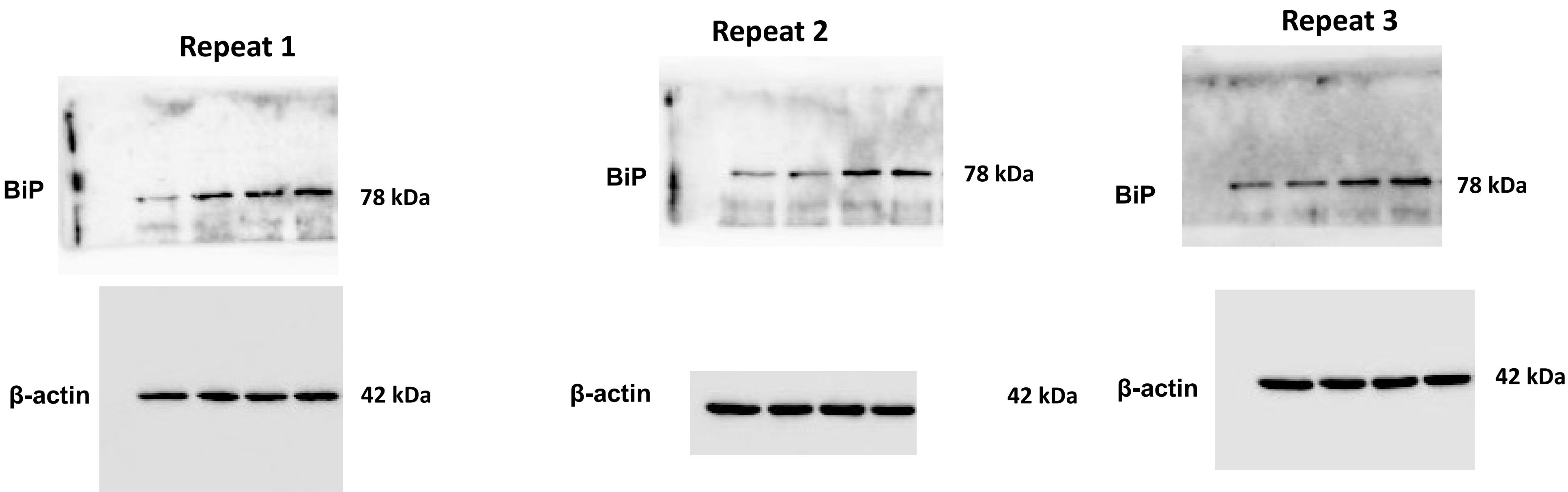
Repeat 1
BiP
78 kDa
β-actin
42 kDa
Repeat 2
BiP
78 kDa
β-actin
42 kDa
Repeat 3
BiP
78 kDa
β-actin
42 kDa

Figure 4A

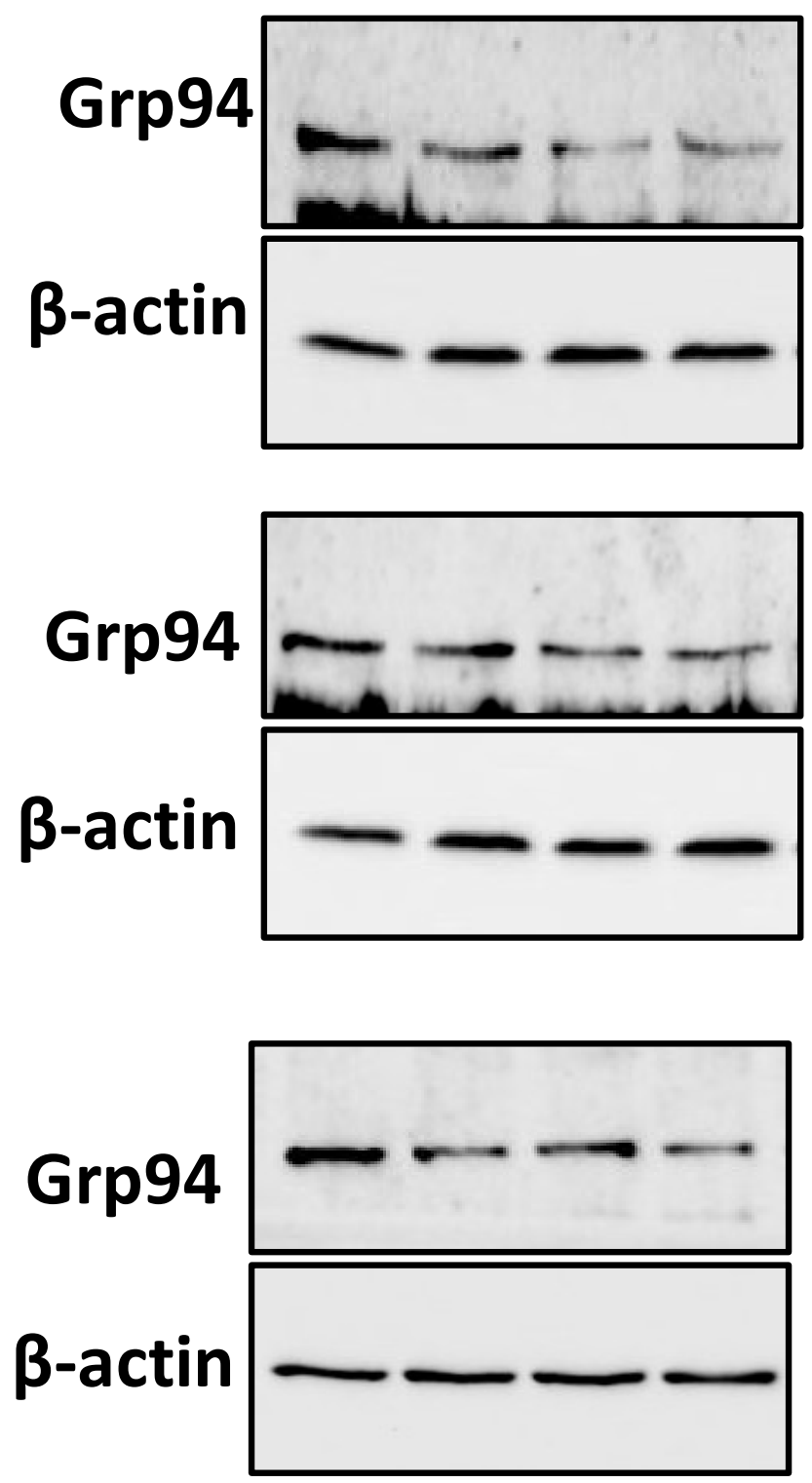
Grp94
β-actin
Grp94
β-actin
Grp94
β-actin

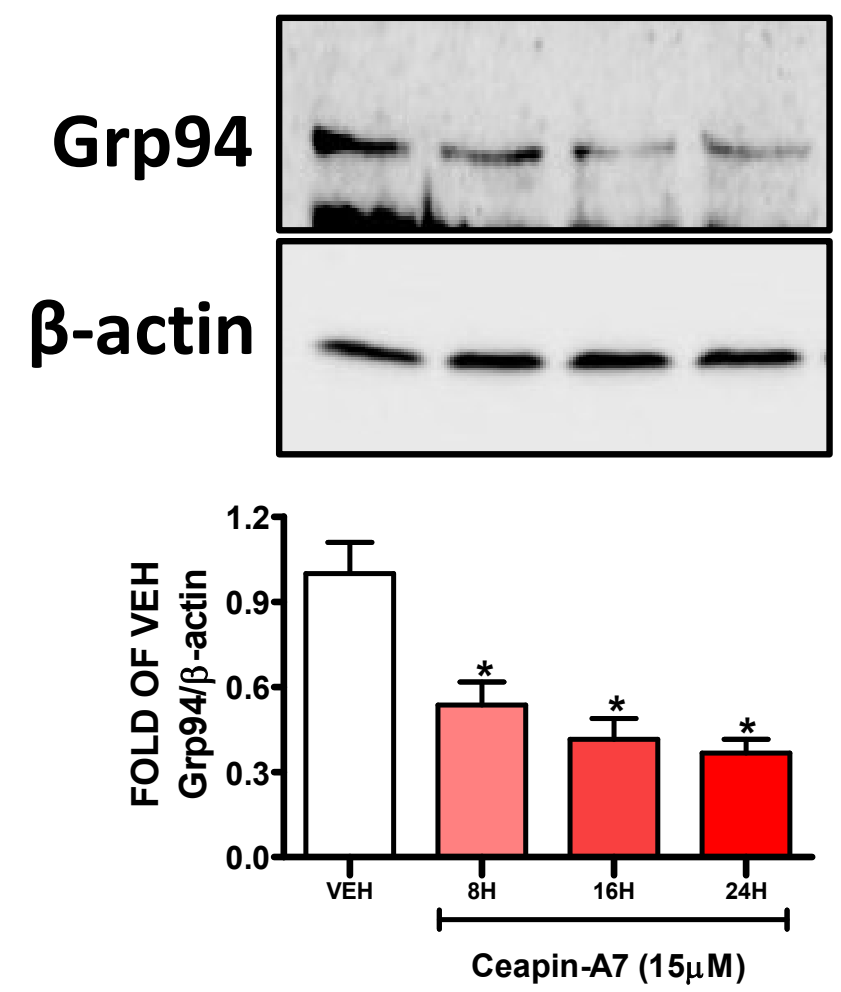
Grp94
β-actin
FOLD OF VEH
Grp94/β-actin
1.2
0.9
0.6
0.3
0.0
VEH
8H
16H
24H
Ceapin-A7 (15μM)

Figure 4A

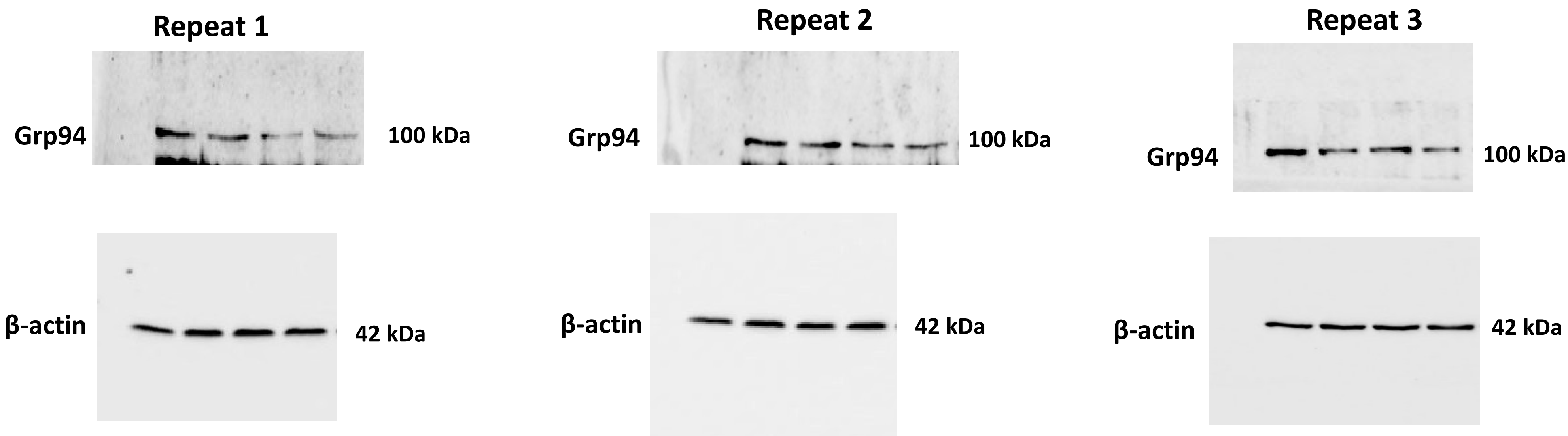

Repeat 1
Grp94
100 kDa
β-actin
42 kDa
Repeat 2
Grp94
100 kDa
β-actin
42 kDa
Repeat 3
Grp94
100 kDa
β-actin
42 kDa

Figure 4B

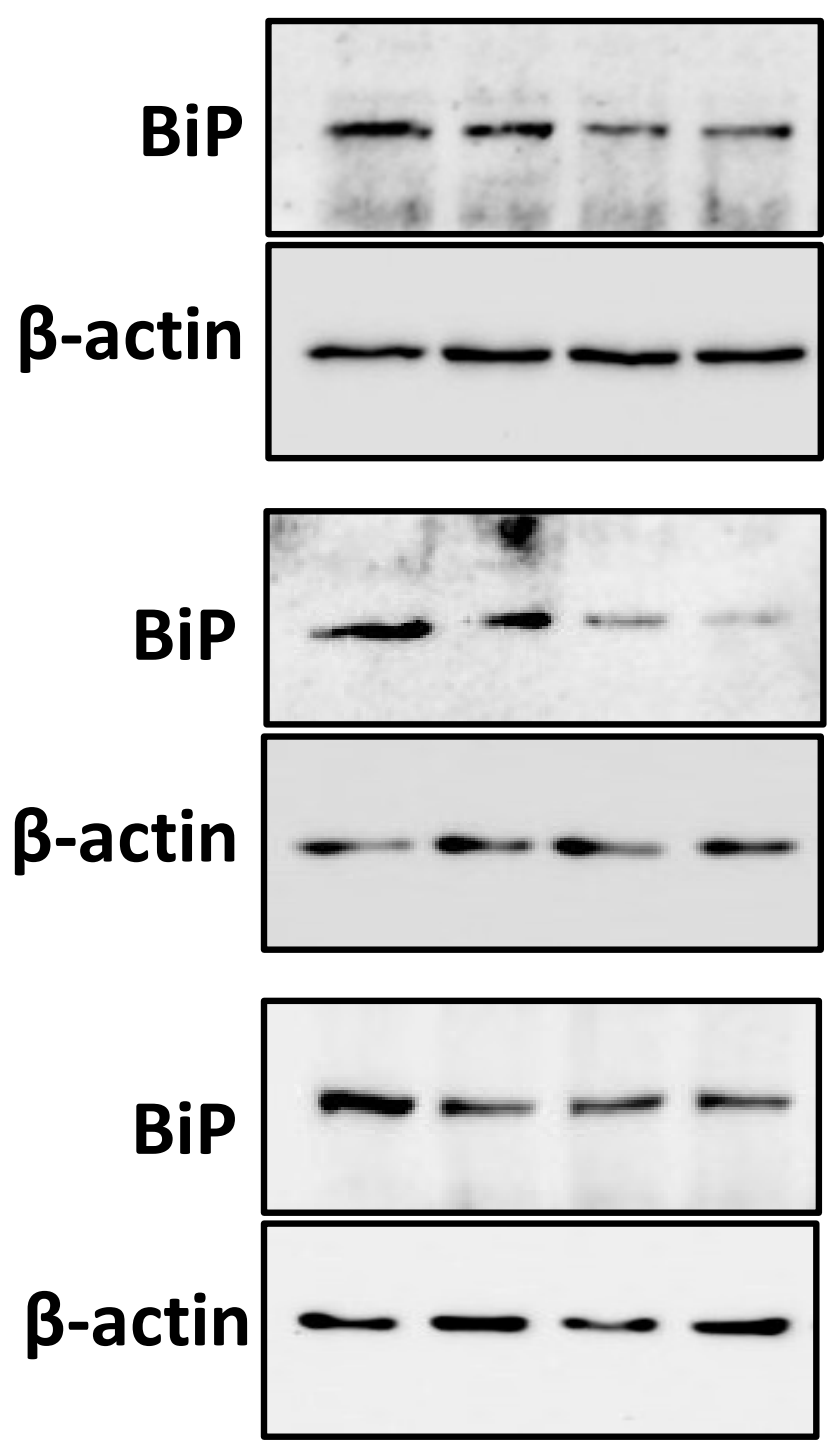

BiP
β-actin
BiP
β-actin
BiP
β-actin

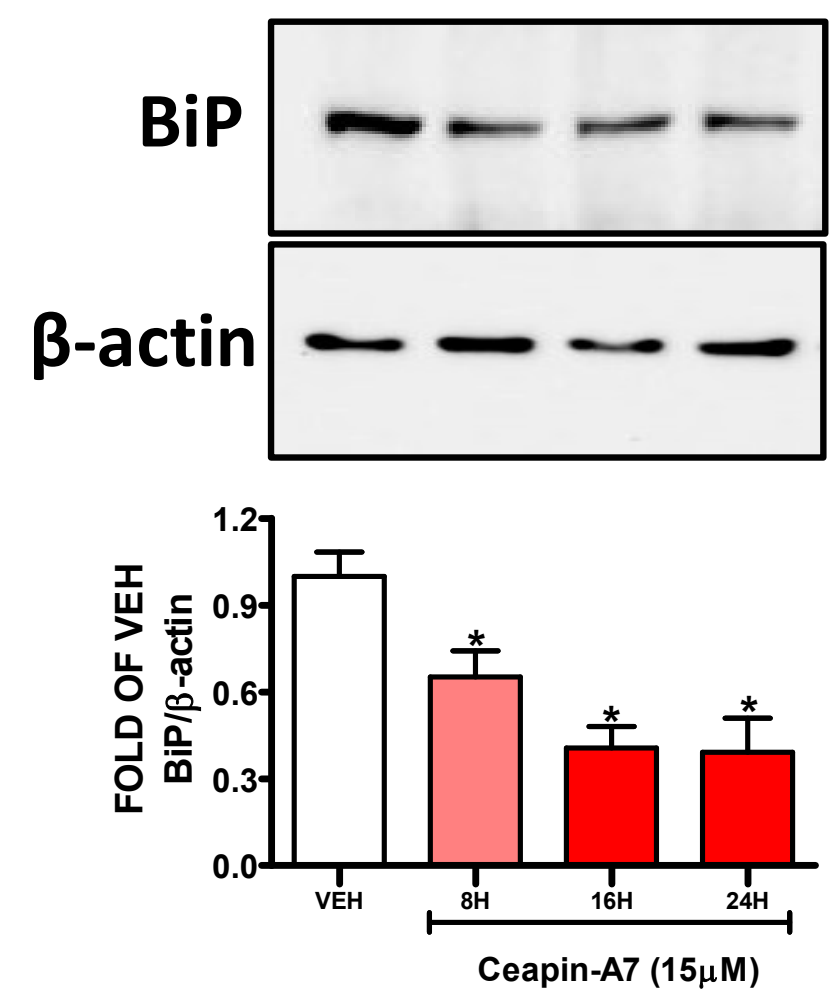

BiP
β-actin
FOLD OF VEH
BiP/β-actin
1.2
0.9
0.6
0.3
0.0
*
*
*
VEH
8H
16H
24H
Ceapin-A7 (15μM)

Figure 4B

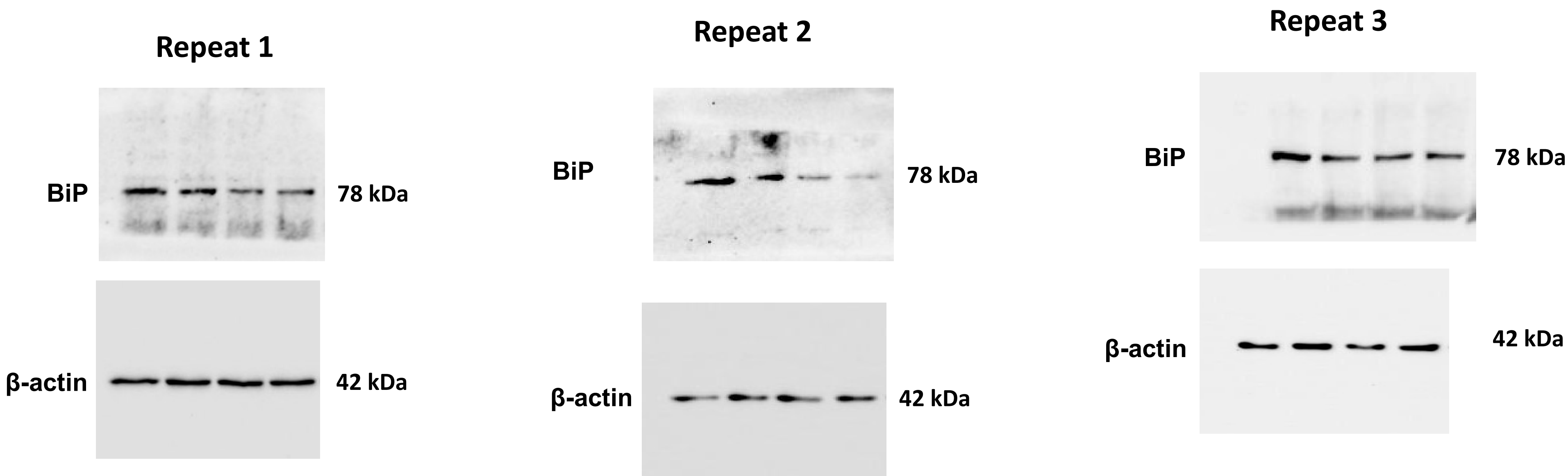
Repeat 1
BiP
78 kDa
β-actin
42 kDa
Repeat 2
BiP
78 kDa
β-actin
42 kDa
Repeat 3
BiP
78 kDa
β-actin
42 kDa

Figure 4C

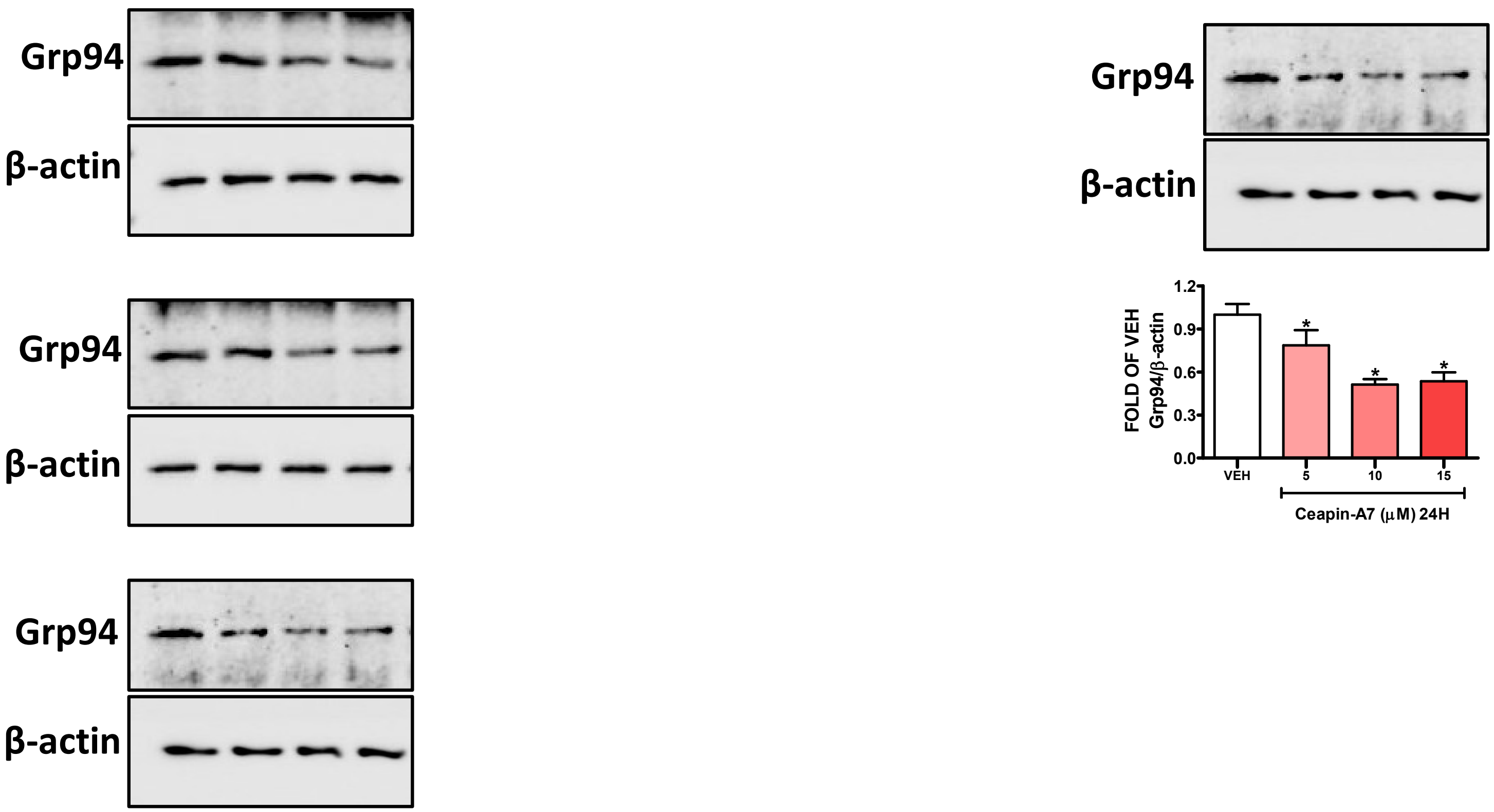

Grp94
β-actin
Grp94
β-actin
Grp94
β-actin
Grp94
β-actin
FOLD OF VEH
Grp94/β-actin
1.2
0.9
0.6
0.3
0.0
*
*
*
VEH
5
10
15
Ceapin-A7 (μM) 24H

Figure 4C

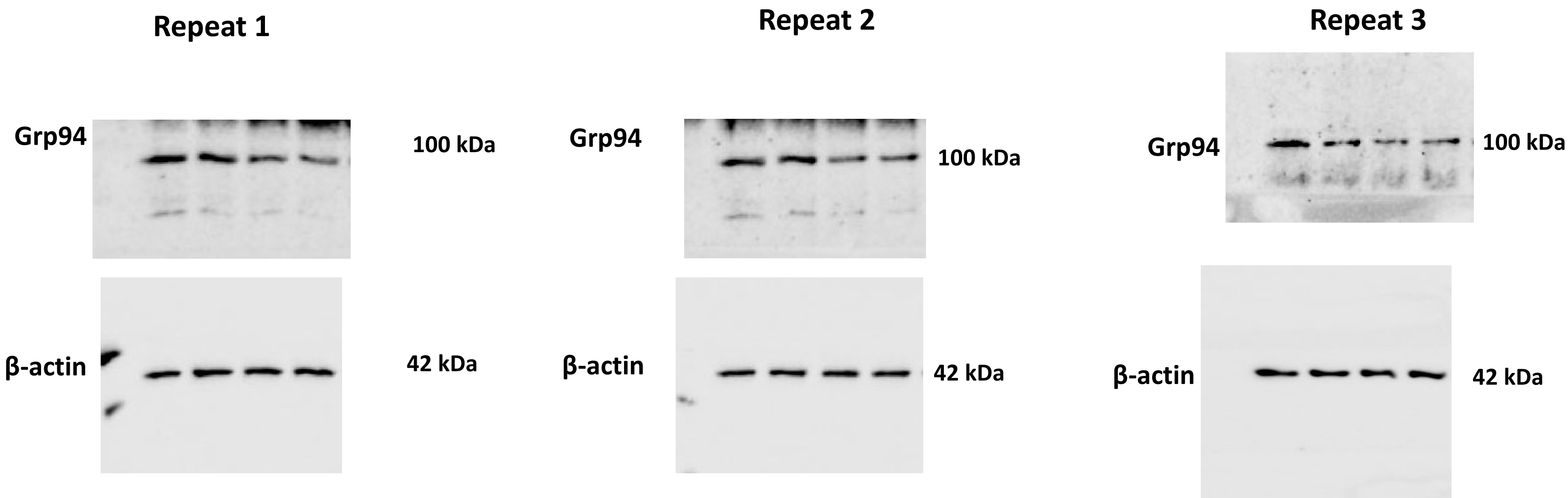
Repeat 1
Grp94
100 kDa
β-actin
42 kDa
Repeat 2
Grp94
100 kDa
β-actin
42 kDa
Repeat 3
Grp94
100 kDa
β-actin
42 kDa

Figure 4D

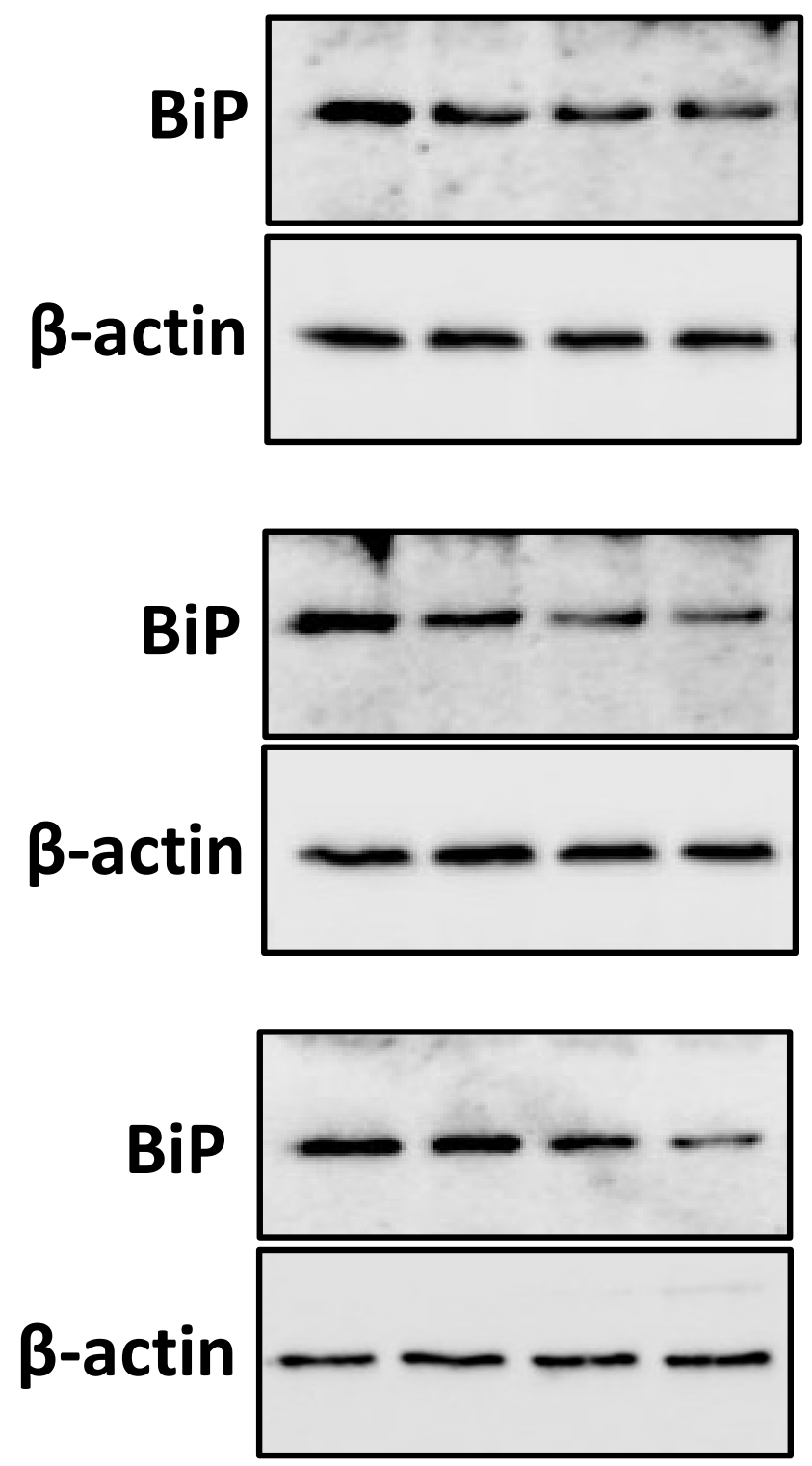

BiP
β-actin
BiP
β-actin
BiP
β-actin

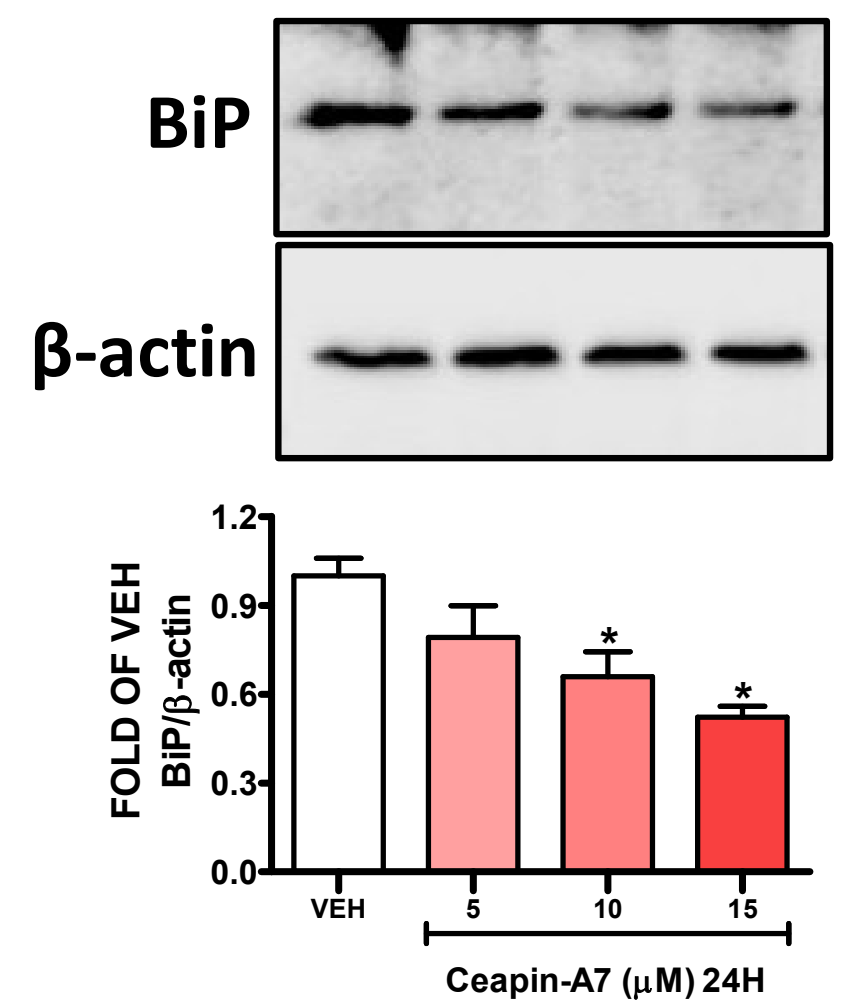

BiP
β-actin
FOLD OF VEH
BiP/β-actin
1.2
0.9
0.6
0.3
0.0
VEH
5
10
15
Ceapin-A7 (μM) 24H

Figure 4D

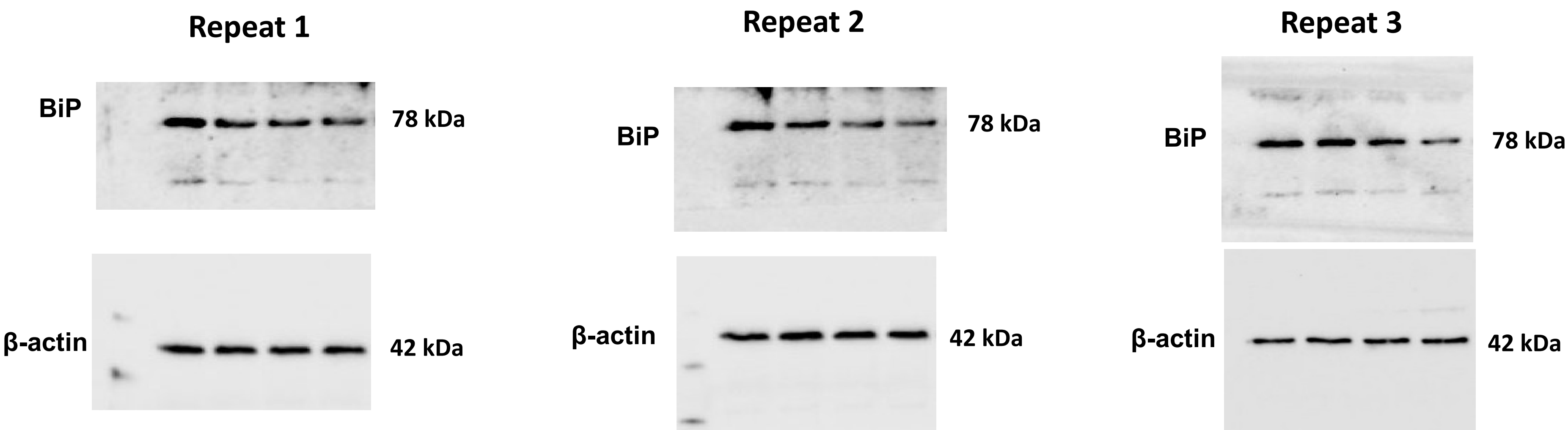

Repeat 1
BiP
78 kDa
β-actin
42 kDa
Repeat 2
BiP
78 kDa
β-actin
42 kDa
Repeat 3
BiP
78 kDa
β-actin
42 kDa
